# Inducing Cancer Cell Killing Using DNA Nanostructure-Mediated Superclustering of Death Receptors

**DOI:** 10.1021/acs.nanolett.5c01122

**Published:** 2025-04-08

**Authors:** Göktuğ Aba, Subinuer Abudukelimu, Margot de Winter, Gabriella Collu, Erik Bos, Sebastiaan M. W. R. Hamers, Lukas J. A. C. Hawinkels, Nadine van Montfoort, Ferenc A. Scheeren, Thomas H. Sharp

**Affiliations:** 1 Department of Cell and Chemical Biology, Leiden University Medical Center, 2333 ZG Leiden, The Netherlands; 2 Department of Gastroenterology and Hepatology, Leiden University Medical Center, 2333 ZG Leiden, The Netherlands; 3 Department of Dermatology, Leiden University Medical Center, 2333 ZG Leiden, The Netherlands; 4 School of Biochemistry, University of Bristol, Bristol, BS8 1TD, United Kingdom

**Keywords:** TRAIL, DNA nanotechnology, Immunology, Death receptor, Cell killing, Nanomedicine

## Abstract

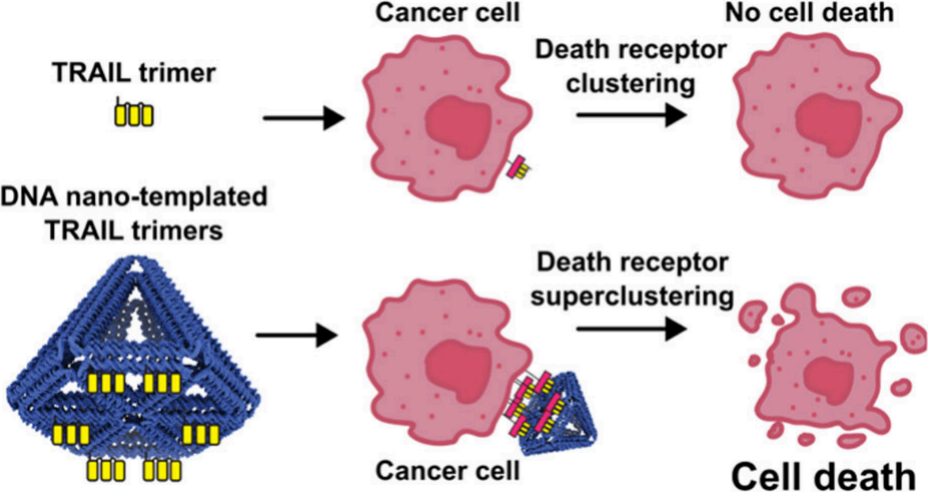

Clustering of type-II tumor necrosis factor receptors (TNFRs) is required to induce intracellular signaling. Current methods for receptor clustering lack precise control over ligand valency and spatial organization, potentially limiting optimal TNFR activation, biological insight, and therapeutic efficacy. DNA nanostructures provide nanometer-precise control over molecular arrangement, allowing control of both ligand spacing and valency. Here, we produce a DNA nanostructure decorated with controlled numbers of engineered single-chain TNF-related apoptosis-inducing ligand (sc-TRAIL) trimers, which bind death receptor 5 (DR5) with native affinity and geometry and enable investigation of the geometric parameters influencing apoptotic pathway activation. We show that cell killing is affected by receptor valency and separation and enhanced by superclustering sc-TRAIL trimers, which can induce cell killing in human primary pancreatic and colorectal cancer organoids. Together, our data show that control of receptor superclustering enhances our understanding of receptor activation mechanisms and informs the development of more effective cancer therapies.

Members of the tumor necrosis factor (TNF) receptor superfamily (TNFRSF) have been extensively studied and targeted for disease treatment due to important roles in cell proliferation, cell death, immune regulation, and morphogenesis.^1^ In particular TNF-related apoptosis inducing ligand (TRAIL) is a well-studied member of the TNF superfamily for its ability to induce apoptosis upon binding death receptors 4 and 5 (DR4/5).^2,3^ The selective ability of TRAIL to induce apoptosis in cancer cells while sparing healthy cells is a promising route for cancer therapy.^4^ However, several antibodies targeting DR4/5 failed to show clinical benefits, likely due to insufficient receptor activation.^5−7^

Receptor clustering is required in order to induce intracellular signaling for many TNFRSF members.^5,8^ In common with other TNFRSF ligands (TNFL), TRAIL forms homomeric trimers that induce DR4/5 trimerization upon binding. These DR clusters may also require further oligomerization, known as superclustering, to induce intracellular signaling and trigger apoptosis,^1,9^ and many agonistic antibodies therefore require additional cross-linking for effective induction of the receptors.^5,10^ It is known that receptor activation can be affected by the distance between ligands and the nanoscale arrangement of the presented ligands.^11,12^ Although peptide-, dextran-, or graphene-based scaffolds have been used to cluster and activate receptors,^13−16^ these scaffolds lack the precision to present ligands at defined sub-nanometer distances.^12,17^ In contrast, DNA origami nanostructures offer precision over ligand arrangement,^12,18−20^ with nanometer control over interligand distances, geometry, and valency.^21^ Although DNA nanostructures have been previously used to study the clustering of DR5 using TRAIL-mimicking peptides and TRAIL protein multimers,^11,12^ the relationship between highly defined DR superclustering and apoptosis induction has not been studied before.

Although antagonistic antibodies, which inhibit or block a specific function, are in clinical use, there are relatively few clinically relevant *agonistic* antibodies.^22^ For certain receptor families, such TNFRs, receptor clustering has been shown to be essential for activation, yet predicting or achieving this clustering remains challenging. Inducing receptor clustering has the potential to target a broad range of activating receptors for clinical development,^23,24^ many of which currently lack viable therapeutic options. Consequently, there is an unmet need for novel technology to facilitate the development of agonistic drugs. Here, we use DNA nanotechnology to nanopattern single-chain TNF-related apoptosis-inducing ligand (sc-TRAIL). This allowed us to study the effect of superclustering of DR5 with different valencies and interligand distances on the killing of cells and organoids. These geometric parameters provide insights into the requirements of receptor superclustering that will be important for developing or improving existing therapeutics.

To provide insights into the geometric constraints of TRAIL-DR5 binding, we analyzed the crystal contacts for various TNFR/TNFL structures in the protein databank (PDB).^25^ We identified a structure that formed closely packed dimers of TNFL trimers separated by ∼6 nm (PDB code 6MGP; Figure 1A).^25^ All known structures of TNFR/TNFL complexes are highly homologous,^26^ and so this distance was presumed to be the smallest distance between TRAIL trimers. Furthermore, applying this dimeric interface to adjacent trimers led to the generation of hexagonal superclusters of TRAIL/DR5, each separated by ∼6 nm (Figure 1A).

**Figure 1:**
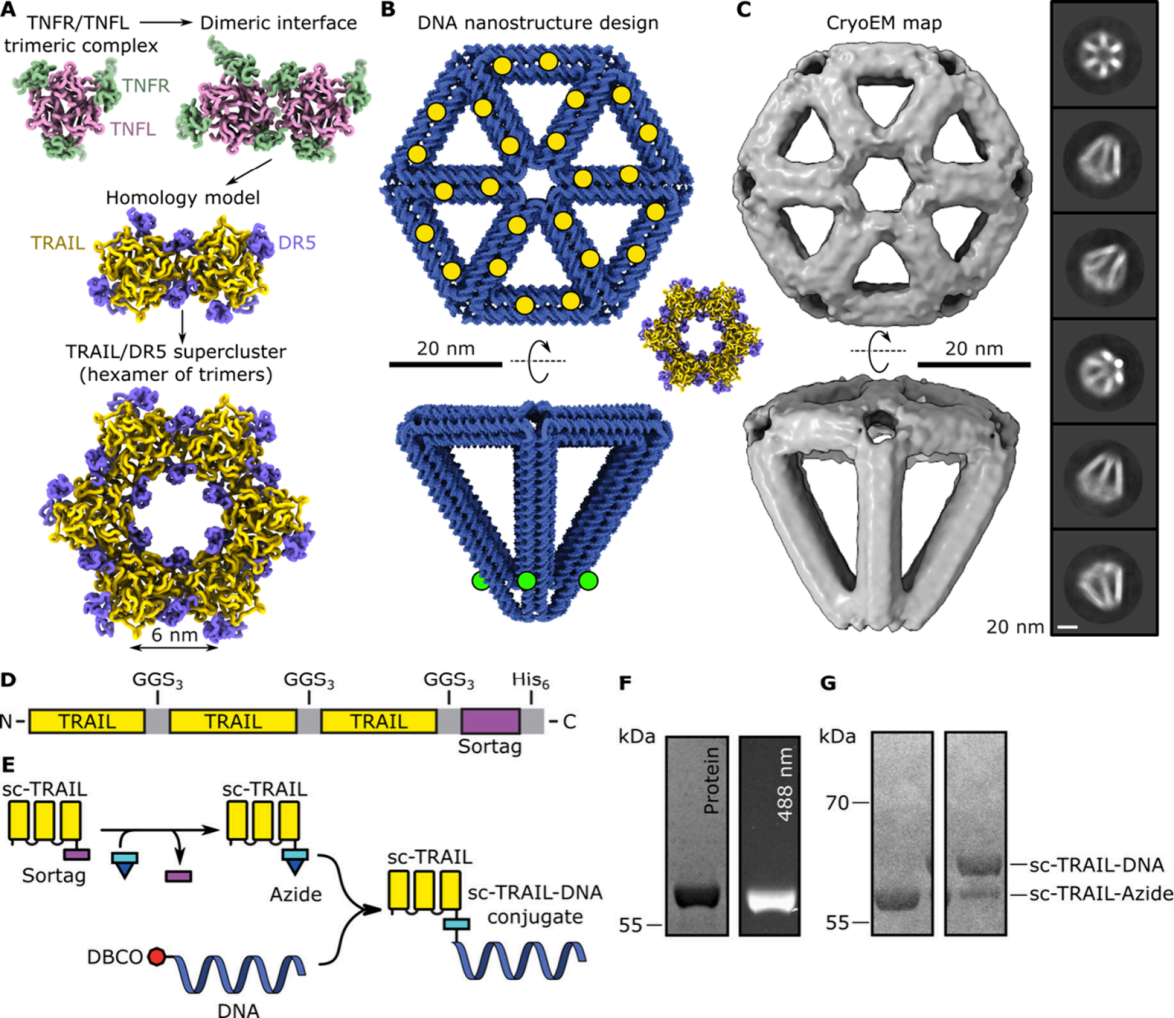
Design of a DR5-templating DNA nanostructure. **A)** Analysis of crystal structures of 4-1BB/L (PDB code 6MGP) reveals a dimeric interface between trimers of 4-1BBL. Homology modeling TRAIL/DR5 (PDB code 1D4V) indicates a model of TRAIL/DR5 trimers separated by 6 nm on a hexagonal lattice. **B)** Design of a DNA nanostructure able to template up to 24 proteins on the hexagonal surface (yellow circles) and up to 6 fluorophores at the pyramid apex (green circles). A model of the TRAIL/DR5 hexamer is shown for scale. **C)** CryoEM map and representative 2D classes of the resulting DNA nanostructure indicate correct folding. **D)** Schematic overview of the sc-TRAIL fusion protein with C-terminal sortag and a His_6_. **E)** Schematic overview of the reactions to attach the DNA handle to sc-TRAIL. **F)** SDS-PAGE of sc-TRAIL attachment to a fluorescent peptide, GGG-K(FITC)-G. Overlap of a fluorescent band with sc-TRAIL confirms the conjugation of the fluorescent peptide. **G)** SDS-PAGE of sc-TRAIL conjugation with the azide-modified peptide, followed by the click reaction with the DBCO-functionalized DNA handle. The shift in the band size confirms that the conjugation of the DNA handle was successful.

This distance and geometry represent the highest possible density of TRAIL/DR5 and may therefore correspond with high receptor activation. To ascertain how the geometry of TRAIL clusters and superclusters affects DR5 signaling, we designed and synthesized a DNA nanostructure able to pattern up to 24 proteins on an extended hexagonal lattice, with a minimal conjugation spacing of 6.6 nm (Figure 1B).

A DNA nanostructure comprising a hexagonal-based pyramid was designed using Athena, as described previously.^27^ This nanostructure comprised a 3D wireframe construct stabilized by six DNA double helices per site, resulting in a rigid structure. Next, 24 sites on the hexagonal face of the nanostructure were identified using UCSF ChimeraX, as well as 6 sites for conjugation of fluorophores close to the apex of the pyramid (Tables S1, S2, S3, S4). The staples corresponding to these conjugation sites were extended with ssDNA handles to enable complementary base pairing of proteins and fluorophores attached to cognate single-stranded DNA after formation of the nanostructure by annealing (see supplementary methods). Gel-electrophoresis was used to assess the folding of the DNA nanostructures (Figures S1A,B). The shift in the band size from the scaffold alone (ssDNA M13m18 genome) represents folding of the DNA nanostructure. From this analysis, the optimal concentrations of MgCl_2_ and NaCl were determined to be 14 mM and 20 mM, respectively. We followed this by confirming the structural integrity using negative-stain transmission electron microscopy (TEM) (Figures S1C,D). The presence of DNA nanostructures with the designed shape verified correct self-assembly. The DNA nanostructures were further characterized using single-particle cryo-electron microscopy (cryoEM) (Figures 1C and S2), which confirmed the designed nanoscale shape of the DNA nanostructure.

To maintain the native binding orientations and affinities for DR5, we engineered a single-chain variant of trimeric TRAIL (sc-TRAIL),^28^ where the three extracellular domains of TRAIL monomers that bind to DR5 (amino acids 110–281; Table S5) were covalently fused and separated by a triple GGS sequence (Figure 1D). A sortag consisting of the peptide sequence LPETGG, which is recognized by the Sortase 5M enzyme, was added to the C-terminus to enable site-specific addition of an azide moiety for downstream conjugation to DNA or other molecules via copper-free click chemistry (Figure 1E). Furthermore, the sortag was followed by a 6-His tag to facilitate purification (Table S5).

Using sortase allowed us to conjugate a single DNA handle, ensuring site-specific modification. The transpeptidation reaction was performed on the recombinant sc-TRAIL to attach either a FITC or an azide. Initially, to optimize the reaction, a FITC fluorophore-containing peptide, with sequence GGG-K(FITC)-G (where the FITC is attached to the lysine side chain), was conjugated to sc-TRAIL. Analysis by SDS-PAGE revealed an intense fluorescent band at the same height as sc-TRAIL, indicating successful conjugation of the FITC peptide to the sc-TRAIL (Figure 1F). Next, we scaled up the reaction to attach the azide (N_3_)-containing peptide, GGG-K(N_3_) (where the azide is attached to the lysine side chain), before purification with size exclusion chromatography. Fractions containing sc-TRAIL-N_3_ were pooled, and a copper-free click reaction with a dibenzocyclooctyne (DBCO−)-containing DNA handle was performed. The DNA handle comprised the reverse complement sequence of handles at the 24 locations on the DNA nanostructure and therefore mediates the binding of the DNA–protein conjugate to the DNA origami nanostructure. An increase in mass of the sc-TRAIL-azide protein indicates conjugation to the DNA (Figure 1G). Based on the intensity of the sc-TRAIL-N_3_ band compared to that of sc-TRAIL-DNA, a reaction yield of 70% was achieved.

We determined whether our DNA origami nanostructures presenting sc-TRAIL with various valencies and spacings were able to induce DR5 signaling and cause cell death. To be able to do this, we used widely available Jurkat cells, which are immortalized T cells, as our cancer cell model. First, the cell viability of Jurkat cells was measured using a commercially available MTT assay kit to study the effect of DNA nanostructure alone or soluble sc-TRAIL, neither of which showed any effect on cell viability with the latter even at concentrations up to 1 μM (Figure S3).

Next, we assessed the cell killing effect of sc-TRAIL-decorated DNA origami nanostructures with varying valencies, from one sc-TRAIL up to six sc-TRAIL on the construct, named as Ori-1, Ori-2, etc. The interligand distance of sc-TRAIL was 6.6 nm, unless otherwise noted. DNA origami control without sc-TRAIL did not have any effect on the cell viability (Figure 2A). Interestingly, even Ori-1 (a single sc-TRAIL on the DNA origami nanostructure) was enough to impact cell viability at nanomolar concentrations. Cell killing increased in correlation with sc-TRAIL valency, in the order of Ori-1, -2, -3, -4, -5, and -6 (Figure 2A). The difference in cell killing became statistically significant with Ori-4, where 4 sc-TRAIL molecules were used to supercluster DR5, compared to Ori-0, the DNA origami control (Figure 2A).

**Figure 2:**
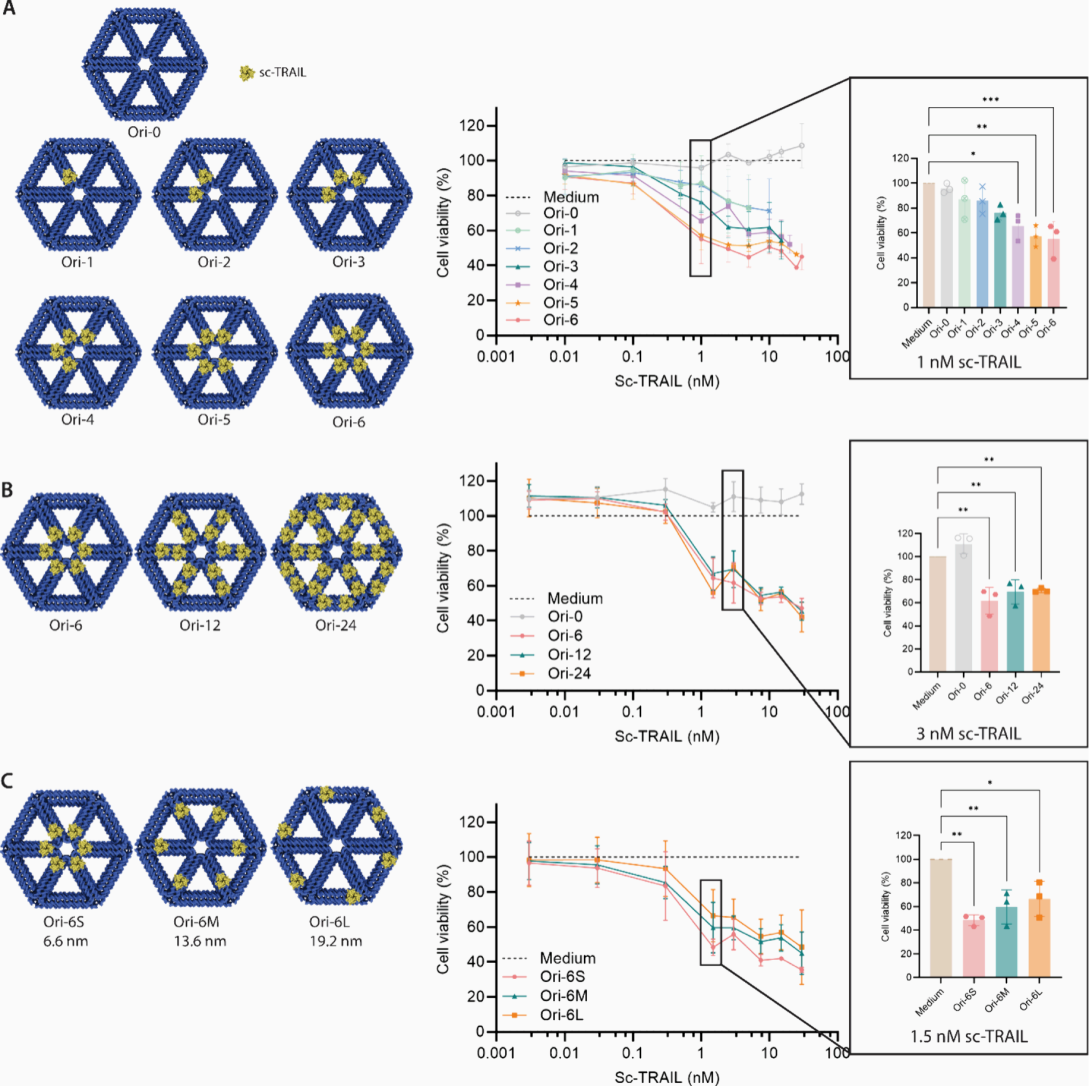
Cytotoxicity of sc-TRAIL-decorated DNA nanostructures in vitro. **A)** Effect of sc-TRAIL valency on Jurkat cell killing. **B)** Further assessment of cell killing effects of 6, 12, or 24 sc-TRAIL-decorated DNA origami nanostructures on Jurkat cells. **C)** Effect of different distancing of sc-TRAIL on Jurkat cell killing. Ori-6S depicts the small distancing of 6.6 nm, Ori-6 M depicts the medium distancing of 13.6 nm, and Ori-6L depicts the large distancing of 19.2 nm between each sc-TRAIL. Data are normalized by taking the medium control as the baseline. Shown data represent the means of three biological replicates with standard deviation (*n* = 3). *p ≤ 0.05, **p ≤ 0.01, ***p ≤ 0.001. One-way ANOVA with Tukey’s correction was used for the statistical analysis.

Our DNA nanostructure was designed to display up to 24 molecules (Figure 1B), and so we then assessed the effect of higher order sc-TRAIL patterns, with 12 and 24 sc-TRAIL (Ori-12 and Ori-24). Although all were able to induce cell killing, we did not observe a difference between DNA origami nanostructures with 6, 12, or 24 sc-TRAIL molecules (Figure 2B). Next, we assessed the effect of different spacing of sc-TRAIL on cell killing with distancing of 6.6 nm (small distancing, Ori-6S), 13.6 nm (medium distancing, Ori-6M), and 19.2 nm (large distancing, Ori-6L), as shown in Figure 2C. Again, these constructs were all able to induce cell killing, although there was a slight advantage to using Ori-6S, with a narrow distance between sc-TRAIL (Figure 2C).

With these results together we can conclude that superclustering indeed has an important role in amplification of DR5 activation. Interestingly, cell killing increased in correlation with sc-TRAIL valency up to six sc-TRAIL trimers on the DNA nanostructure, thereby superclustering up to 18 DR5s. Increasing the sc-TRAIL valency to 12 and 24 did not increase the cell killing further, indicating the limits of the superclustering effect. This limit could be caused by the saturation of the intrinsic signaling pathway where the intracellular signaling pathway components are fully activated and further activation of the receptors does not amplify the response. On the other hand, sc-TRAIL spacing has moderate effects on the cell killing, indicating that spacing between sc-TRAIL trimers is not as impactful as the valency. Considering that multiple DNA nanostructures displaying sc-TRAIL can bind the same cell adjacently, a large distancing construct brings some limitations, since the distance between sc-TRAIL trimers on two adjacent DNA nanostructures may not be 19.2 nm. To be able to study the effects of distancing on superclustered DR5s, further research is needed with larger DNA nanostructures or where the distance between the adjacent DNA nanostructures is controllable.

To determine whether cell killing was indeed due to DR5 binding and activation, we next used the Ori-6S construct on Jeko-1 cells that had genes in the DR pathway genetically knocked out (KO), specifically, DR5 and aspartate-specific cysteine protease (caspase)-8.^29^ Programmed cell death mediated by DRs induces activation of intracellular caspase-8.^30^ Jeko-1 cells, derived from B cells, are also a different model cell line to Jurkat cells, and therefore provide a more robust analysis of the applicability of our superclustering DNA nanotemplate. Ori-6S showed cell killing on wild type (WT) JeKo-1 cells (Figure S4). Importantly, cell killing by Ori-6S was diminished in DR-5 KO and completely abrogated with caspase-8-KO cells (Figure S4). Although DR4 has been previously shown to function in the absence of DR5,^31^ we presume that the relatively low DR4 expression in both protein and mRNA levels in the JeKo-1 cells, compared to DR5,^29^ is the cause of the rescue for cell killing in the DR5 KO cells. Together, these KO data indicate that the sc-TRAIL-decorated nanostructures induce cell death mediated by the DR pathway.

Pancreatic ductal adenocarcinoma (PDAC) is a malignancy with very low 5-year survival rate due to limited treatment options at time of diagnosis. As a consequence of the high incidence, colorectal cancer (CRC) has one of the highest number of cancer-related deaths in the US.^32^ Therefore, better treatment options are urgently needed for these diseases. Having optimized the cell-killing potential of sc-TRAIL-DNA-origami nanostructures, we examined the efficacy of Ori-6S in patient-derived PDAC and CRC-derived organoids (Figure 3A). PDAC organoids were exposed to varying concentrations of sc-TRAIL (0.03, 0.3, 3, and 30 nM) or Ori-6S (0.03, 0.3, 3, and 30 nM) for 4 days. Bright-field images were taken daily to monitor changes in organoid morphology (Figure S5A). Organoids treated with 30 nM Ori-6S accumulated dead cells in their lumens (white arrows, Figure 3B), whereas sc-TRAIL-treated organoids expanded and retained their clear cystic structure. The origami control resembled the medium control, displaying increased organoid size over time and no accumulation of dead cells in the lumen. A minor amount of cell debris was observed in the lumen of larger organoids treated with the mix of DNA origami plus sc-TRAIL-sortag (unconjugated sc-TRAIL control), possibly due to organoid overgrowth during the experiments. Quantification of cell viability revealed that Ori-6S induced significantly more cell death compared to unconjugated and sc-TRAIL controls (Figure 3C). Notably, even at a concentration as low as 3 nM, Ori-6S induced significant cell death in PDAC organoids, demonstrating its superior cell-killing ability (Figure S5B).

**Figure 3:**
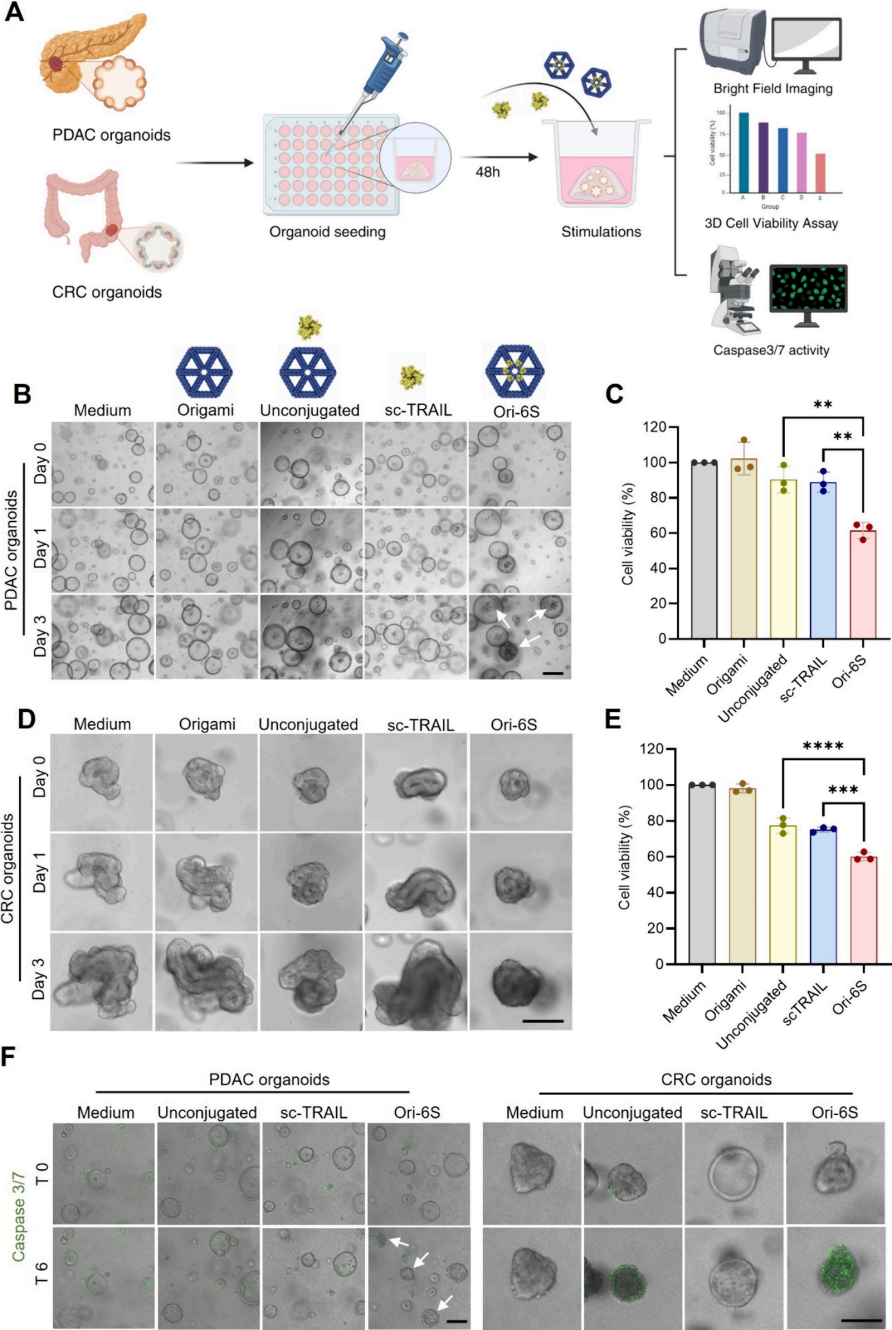
Ori-6S induces higher cell death in primary pancreatic and colorectal cancer organoids. **A)** Schematic overview of the patient-derived organoid experiments. Created in BioRender. Aba, G. (2025) https://BioRender.com/4lrtce9. Representative bright-field (BF) images of PDAC organoids **(B)** and CRC organoids **(D)** at days 0, 1, and 3 upon treatment with either growth medium only, origami only, 30 nM sc-TRAIL-sortag plus DNA origami mixture (unconjugated TRAIL control), 30 nM scTRAIL, or 30 nM Ori-6S. Scale bar, 100 μm. Cell viability of PDAC organoids **(C)** and CRC organoids **(E)** was measured with CellTiter-Glo 3D assay following 4 days of treatment with the respective conditions. *N* = 3, independent experiments, each with 3 technical replicates. Data are presented as means ± SD, **p ≤ 0.01, ***p ≤ 0.001, ****p ≤ 0.0001 determined by one-way ANOVA with correction for multiple testing (Dunnett’s test) comparing to the medium only control group. **F)** Representative merged BF and fluorescence confocal microscopy images of Caspase3/7-positive organoid cells at 0 and 6 h upon treatment with the respective conditions. Apoptotic cells were stained using the CellEvent Caspase-3/7 detection reagent. Scale bar, 100 μm.

Encouraged by these results, we proceeded to test Ori-6S in CRC organoids. Unlike PDAC organoids, CRC organoids formed more compact crypt-like structures. Increased crypt-like structures were observed in medium-only, origami-only, and unconjugated control groups (Figure 3D). Both sc-TRAIL and Ori-6S treatments showed disrupted organoid morphology by day 3, with sc-TRAIL-treated organoids growing larger over 2 days compared to Ori-6S-treated organoids. Additionally, Ori-6S-treated organoids exhibited significantly reduced cell viability compared to unconjugated and sc-TRAIL controls (Figure 3E).

To determine if the observed cell death was due to TRAIL-induced apoptosis, we visualized caspase 3/7 activity using live-cell confocal microscopy (Supplemental Movies 1 and 2). Both PDAC and CRC organoids were exposed to a caspase-dependent reporter during treatment. After 6 h, Ori-6S-treated PDAC organoids displayed reduced size, disrupted morphology, and abundant caspase 3/7-positive cells in the lumen (Figure 3F, Supplemental Movie 1). Similar effects were seen in CRC organoids treated with Ori-6S at 6 h (Figure 3F, Supplemental Movie 2), showing numerous green caspase 3/7-positive cells in the center, confirming caspase 3/7 activation. In contrast, only few caspase 3/7-positive cells were observed in the medium control, unconjugated, and sc-TRAIL-treated groups. Although the number of apoptotic organoid cells was similar among unconjugated, sc-TRAIL, and Ori-6S treatments at 24 h (Figures S6 and S7), Ori-6S induced earlier apoptosis in both PDAC and CRC organoids compared to sc-TRAIL and unconjugated controls, likely due to enhanced clustering of death receptors.

Current approaches to generate therapeutics prioritize high-affinity binders to activate immune receptors. However, high affinity binders may not be essential for effective receptor activation. Low-affinity agonistic antibodies showed stronger agonism compared to high-affinity agonistic antibodies,^33^ and anti-DR5 antibodies have not found use in the clinic.^5−7^ Although high-affinity TRAIL-mimicking peptides clustered on DNA nanostructures have been previously used to study the effects of ligand arrangement on DR activation,^12^ these bind monomerically to DR5 and therefore induce clustering, and not superclustering, as in this study. Here we can bind and pattern up to 24 homotrimeric sc-TRAIL molecules, corresponding to 72 DR5 monomers. Additionally, using TRAIL trimers allows us to maintain the native binding affinity, which is much lower than the TRAIL-mimicking peptides. For the first time, our results have shown that superclustering of ligands defines the effectivity of cell killing, which reveals that 6 TRAIL trimers spaced 6.6 nm apart is optimal to induce cell killing in cells and patient-derived organoids. Such ligand arrangement offers tractable and tunable activation of DR5. This approach enables the determination of optimal ligand–receptor arrangements to deliver the desired receptor activity required for medical translation. The biocompatibility of DNA origami nanostructures is being studied extensively to establish DNA nanostructures as a feasible and safe drug delivery platform,^34,35^ although further research is needed to establish their immunogenicity.^35,36^ Together, these data illustrate the important role that superclustering can play in activation of DRs using sc-TRAIL-decorated DNA origami nanostructures. Interestingly, superclustering is also important in other TNFRs, and a similar approach could therefore be applied to other TNFRs, such as 4-1BB and CD27. Consequently, more detailed understanding of the biology and geometric requirements of receptor superclustering and activation may lead to the development of better, more effective therapeutics.

Here we present data showing that we can regulate death receptor superclustering resulting in activation of the DR pathway using DNA origami nanostructures decorated with TRAIL. This method allowed us to study and understand the nanoscale spatial organization of the death receptors and the effects of superclustered sc-TRAIL ligands with different distancing and valencies. Moreover, utilizing sc-TRAIL allowed us to supercluster DR with native affinity, valency, and geometry. Our results showed that when sc-TRAIL is in solution, it is unable to induce apoptosis in Jurkat cells. However, when the sc-TRAIL was loaded on the DNA nanostructures, cell killing was observed even at nanomolar concentrations. Furthermore, when the valency of the sc-TRAIL was increased, the cell killing effect also increased up to 6 sc-TRAIL ligands, corresponding to 18 DR5 molecules. Surprisingly, when more than 6 sc-TRAIL ligands are loaded on the nanostructure, the increasing effect in cell killing did not change, indicating that no additional intracellular signaling occurs above engagement of 18 DR5 molecules. Using DNA nanostructures to control the ligand separation indicated that cell killing was affected minimally when the distance between the 6 sc-TRAIL ligands was altered from 6.6 nm to 13.6 and 19.2 nm, with 6.6 nm distancing showing optimal cell killing ability. Optimized conditions were then tested on PDAC and primary CRC organoids. Sc-TRAIL-decorated nanostructures showed potent cell killing in both of the organoid models. Using nanopatterned sc-TRAIL to supercluster death receptors provides valuable insights into their activation and offers a potential pathway for the development of novel therapeutics or enhancement of existing treatments. For example, superclustering may play an important role in treating tumors that are resistant to soluble TRAIL and may explain the failure of previous clinical trials targeting DRs.^37,38^ The geometric parameters identified in this study, along with the significance of superclustering, should be considered in the development of targeted therapies for DR.

## Supporting Information Available

PDAC movie: 62 h time lapse movie of Caspase3/7-positive pancreatic ductal adenocarcinoma (PDAC) organoids treated with the respective conditions (MOV)
CRC movie: 24 h time lapse movie of Caspase3/7-positive colorectal cancer (CRC) organoids treated with the respective conditions (MOV)
Structural characterization of DNA nanostructures; supporting cell and organoid killing data; DNA and protein sequences; methods; supplementary references (PDF)

## Supplementary Material

nl5c01122_si_001.mov

nl5c01122_si_002.mov
