# Supplementary material for: Inducing Cancer Cell Killing Using DNA Nanostructure-Mediated Superclustering of Death Receptors
Source: Nano Lett. 2025 Apr 8;25(15):6310–7. doi: 10.1021/acs.nanolett.5c01122 (PMC12007100; doi:10.1021/acs.nanolett.5c01122)
Supplement: Supplementary file 3 — nl5c01122_si_003.pdf [file nl5c01122_si_003.pdf]

Supplementary Information for

Inducing cancer cell killing using DNA nanostructure-mediated superclustering of death-receptors

Göktuğ Aba[1], Subinuer Abudukelimu[2], Margot de Winter[1], Gabriella Collu[1], Erik Bos[1], Sebastiaan M.W.R. Hamers[1], Lukas J.A.C Hawinkels[2], Nadine van Montfoort[2], Ferenc A. Scheeren[3,*] & Thomas H. Sharp[1,4,*]

[1]Department of Cell and Chemical Biology, Leiden University Medical Center, 2333 ZG Leiden, The Netherlands

[2]Department of Gastroenterology and Hepatology, Leiden University Medical Center, 2333 ZG Leiden, The Netherlands

[3]Department of Dermatology, Leiden University Medical Center, 2333 ZG Leiden, The Netherlands

[4]School of Biochemistry, University of Bristol, Bristol, BS8 1TD, United Kingdom

Corresponding authors:

Dr. Ferenc A. Scheeren, f.a.scheeren@lumc.nl

Dr. Thomas H. Sharp, t.sharp@bristol.ac.uk

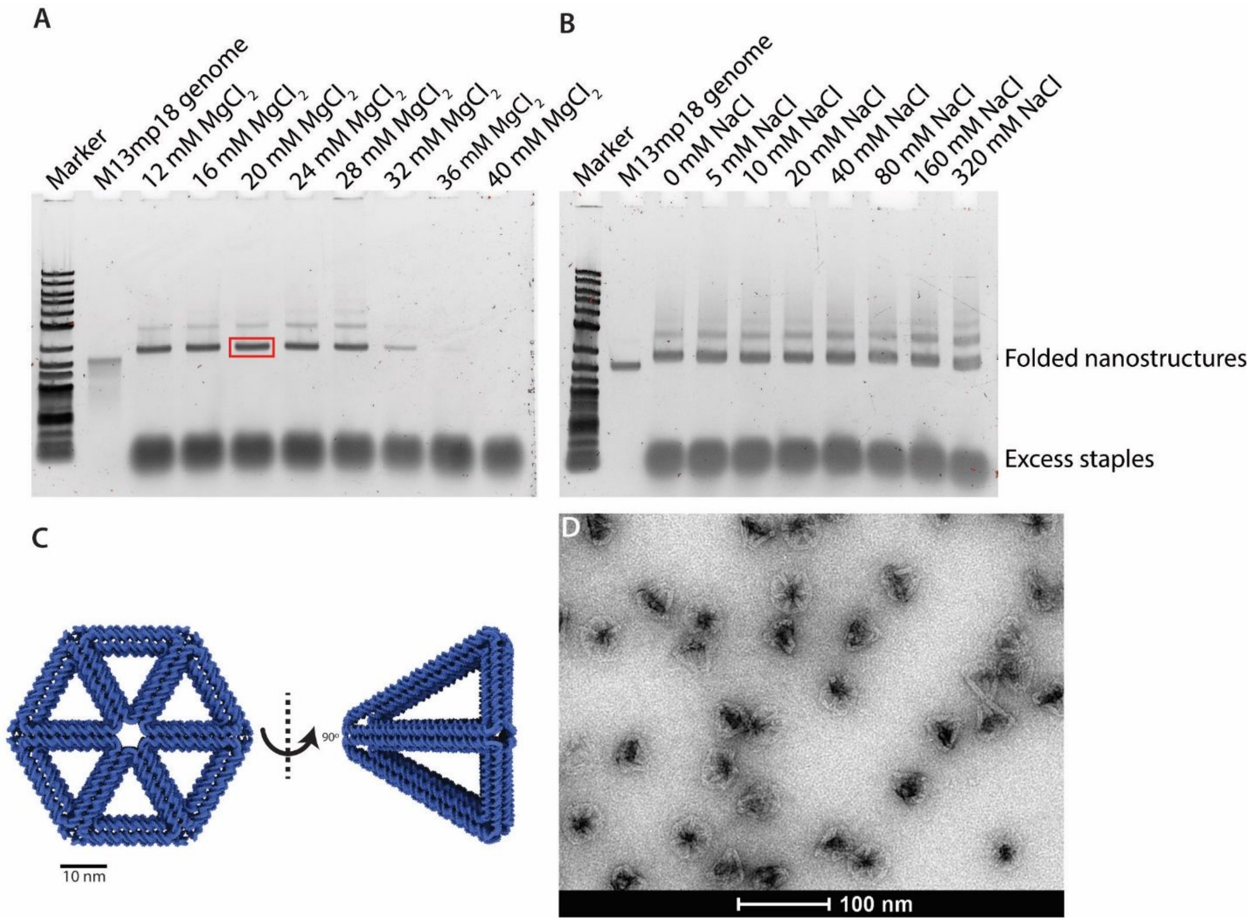

Figure S1. Optimization and characterization of the hexagonal-based pyramid DNA nanostructure. A) *Agarose gel analysis of the optimization of the folding reaction with various $MgCl_2$ concentrations.* ***B)*** *Agarose gel analysis of the optimization of the folding reaction with various NaCl concentrations. Folded nanostructures and the excess staples are indicated. 1% agarose gel was run at 100 V for 1 hour with 3 µL samples that were diluted 12.5x in MQ and loading dye.*[1] ***C)*** *Schematic representation of the pyramid-like DNA origami nanostructure. Scale bar: 10 nm.* ***D)*** *Transmission electron microscopy images of the folding reaction with 20 mM $MgCl_2$ and 5 mM NaCl. Images were obtained with negative staining after purifying the sampels. Scale bar: 100 nm.*

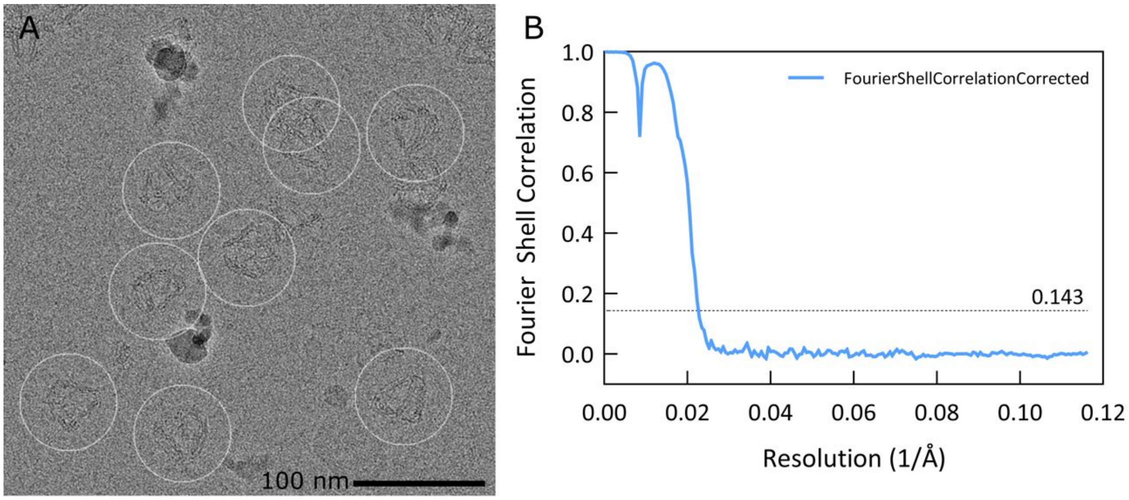

Figure S2. CryoEM characterization of the hexagonal-based pyramid DNA nanostructure. A) *Representative cryoEM image.* ***B)*** *Fourier shell correlation (FSC) curve showing a resolution of 45 Å at FSC=0.143.*

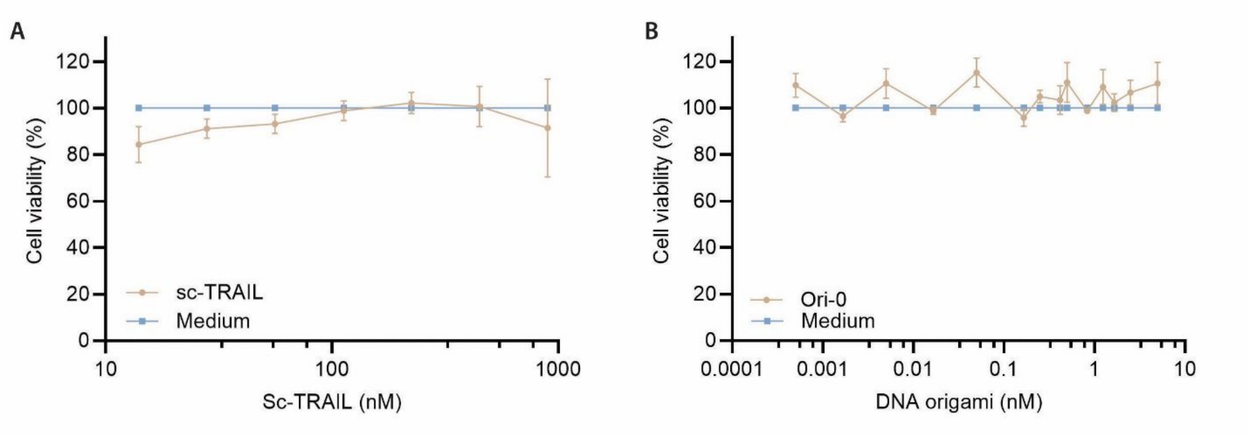

Figure S3. Effect of soluble sc-TRAIL and DNA origami nanostructure alone on Jurkat cell killing. A) *Effect of soluble sc-TRAIL on Jurkat cells compared to medium control.* ***B)*** *Effect of DNA origami nanostructures on Jurkat cells.*

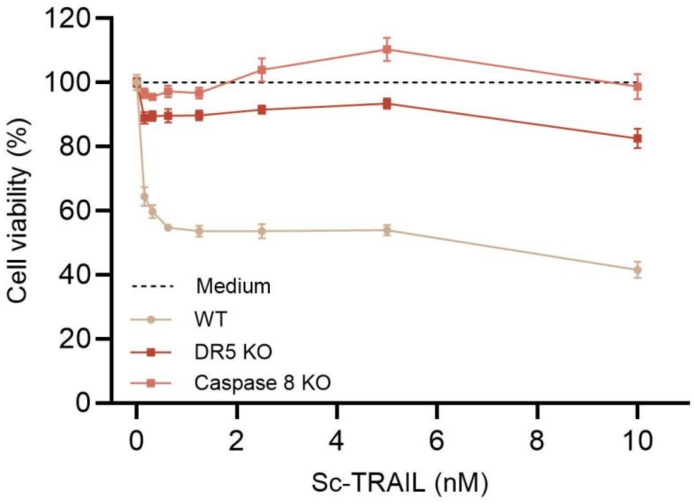

Figure S4. Assessing receptor specificity of Ori-6S construct using death receptor 5 (DR5) and Caspase 8 gene knockout (KO) JeKo-1 cells. *JeKo-1 wildtype (WT), DR5 KO and caspase 8 KO cells were treated with different concentrations of sc-TRAIL on the Ori-6 construct, followed by the determination of the cell viability using the MTT assay. Sc-TRAIL concentration of 10 nM corresponds to 1.6 nM of Ori-6S concentration.Data are normalized by taking the medium control as the baseline. Shown data represents three biological replicates.*

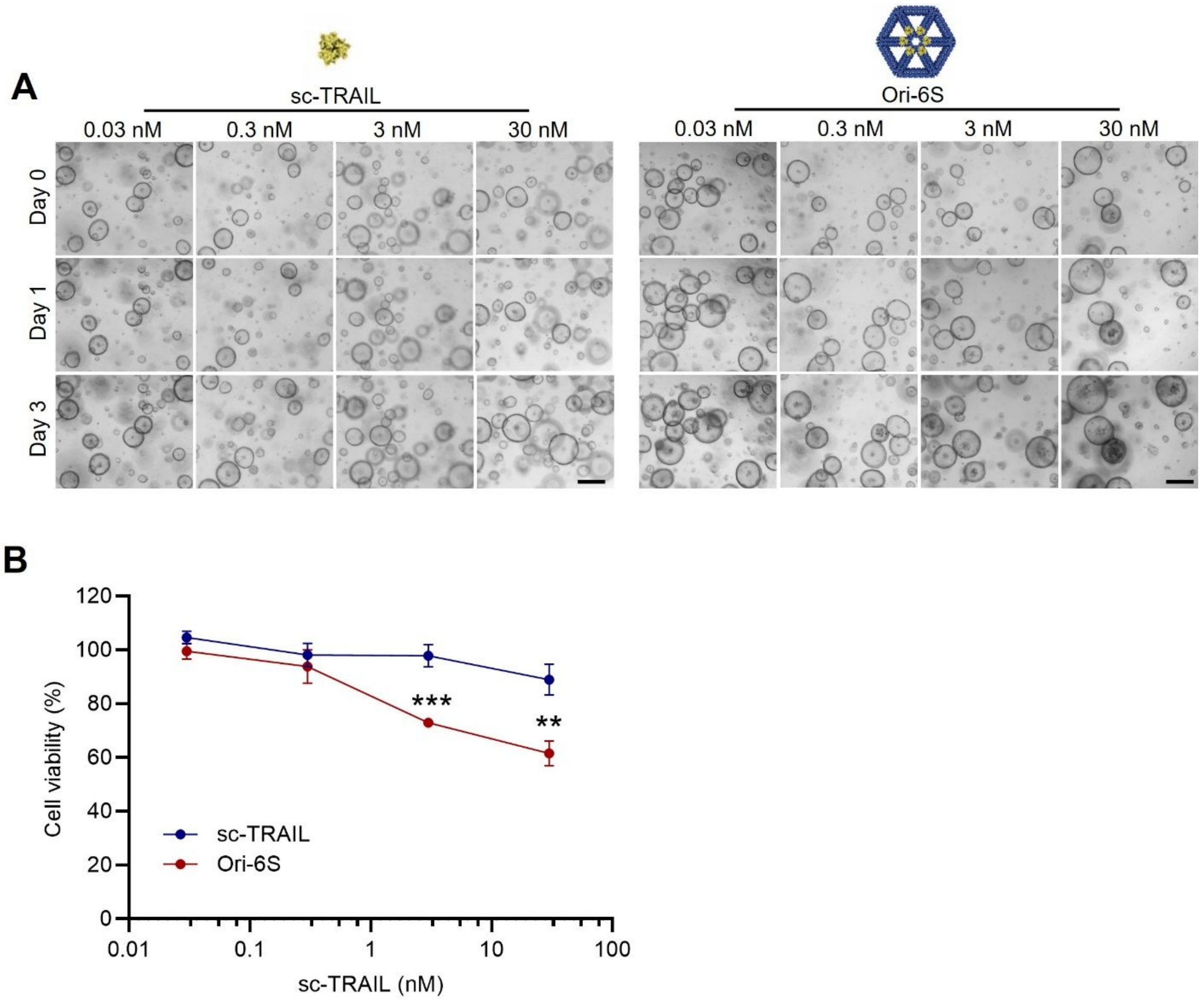

Figure S5. Treatment of patient-derived PDAC organoids with free sc-TRAIL or Ori-6S. A) *Representative BF images of PDAC organoids at days 0,1 and 3 upon treatment with varying concentrations of sc-TRAIL (0.03, 0.3, 3, and 30 nM) or Ori-6S (0.03, 0.3, 3, and 30 nM), respectively.* ***B)*** *Cell viability of PDAC organoids treated with different concentrations of sc-TRAIL or Ori-6S at days 4. ** p≤0.01, *** p≤0.001 determined by one-way ANOVA with correction for multiple testing (Dunnett's test).*

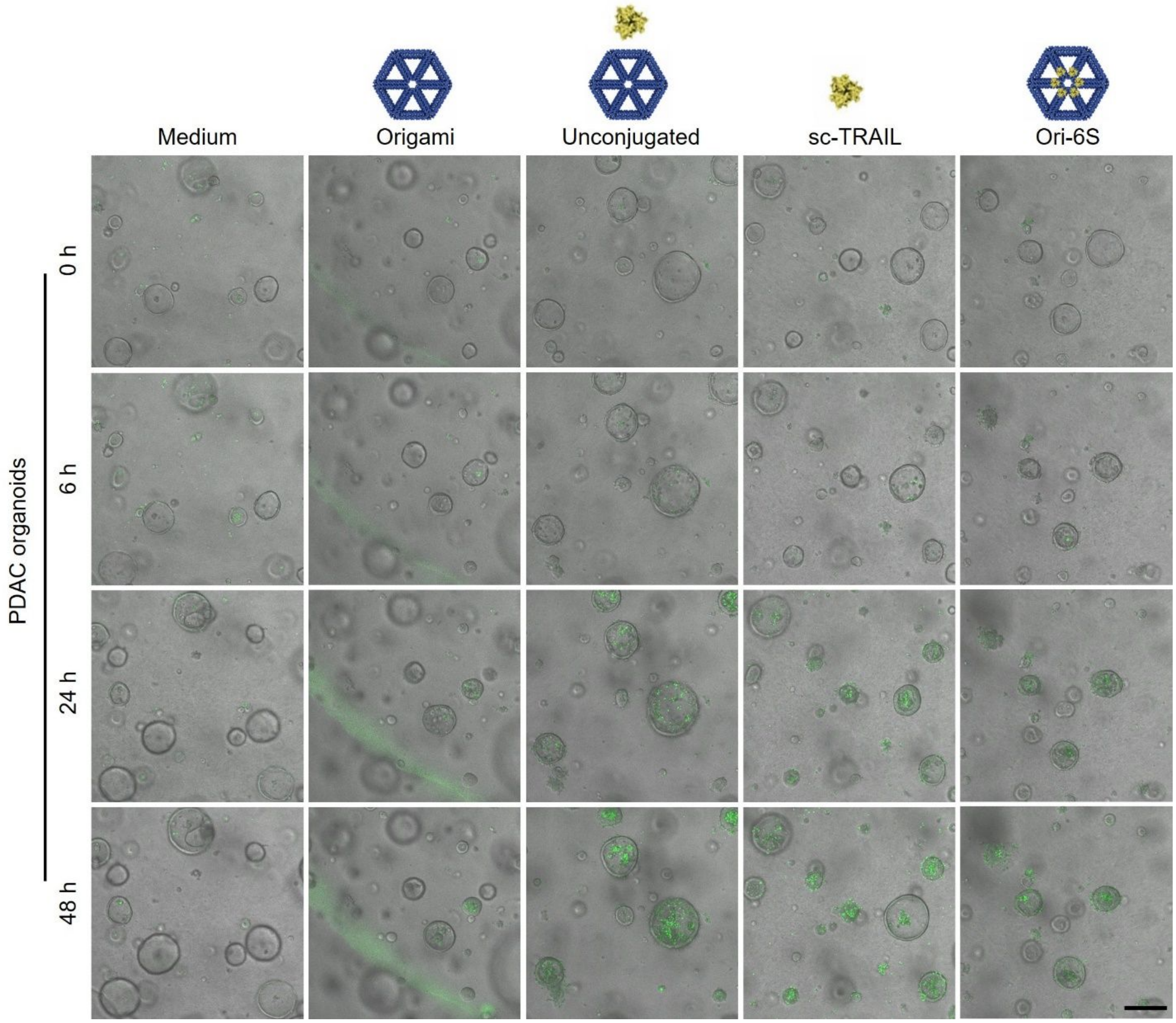

Figure S6. Caspase3/7 activity in PDAC organoids treated with sc-TRAIL or sc-TRAIL decorated DNA nanostructures using fluorescence confocal microscopy. A) *Representative merged BF and fluorescence confocal microscopy images of Caspase3/7-positive PDAC organoid cells at 0, 6, 24, 48 hours upon treatment with either growth medium only, origami only, unconjugated TRAIL, 30 nM scTRAIL or 30 nM Ori-6S. Scale bar, 100 µm.*

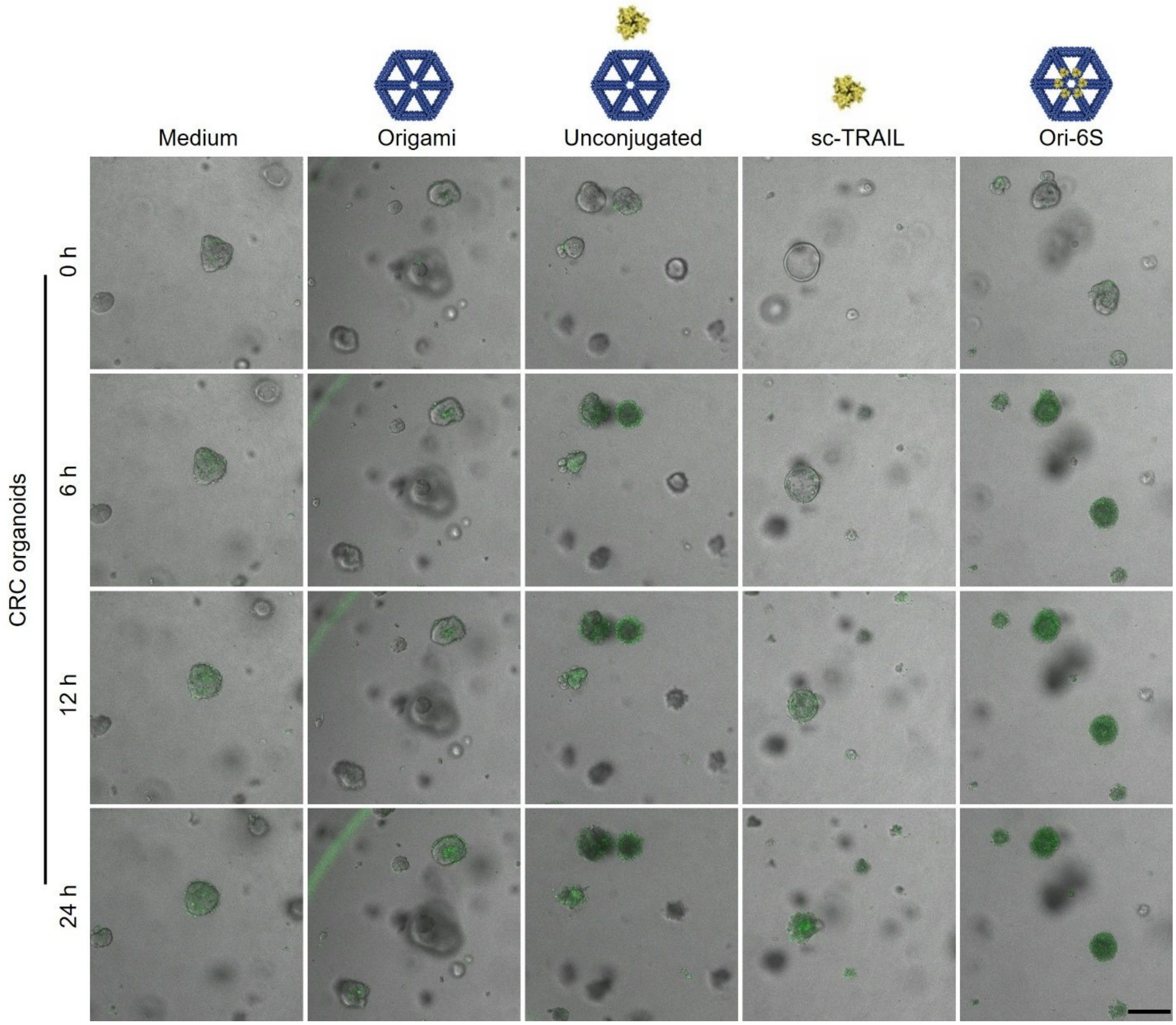

Figure S7. Caspase3/7 activity in CRC organoids treated with sc-TRAIL or sc-TRAIL decorated DNA nanostructures using fluorescence confocal microscopy. A) *Representative merged BF and fluorescence confocal microscopy images of Caspase3/7-positive CRC organoid cells at 0, 6, 12, 24 hours upon treatment with either growth medium only, origami only, unconjugated TRAIL, 30 nM scTRAIL or 30 nM Ori-6S. Scale bar, 100 µm.*

Table S1. Core staples that facilitate the folding of the DNA origami nanostructure.

Name	Sequence
Core_01	TGATTTTTTTTTTTTGAATAC
Core_02	GGGTCTTTTTTTGGTGCCGTA
Core_03	AAAAATCCAGAATCGGGATTT
Core_04	GCGCGTAGAACAAGTAGGGTT
Core_05	ATTGCTTTTTTTTGCGCTCAC
Core_06	ATGGCTTTTTTTTTAGTCTTT
Core_07	AACAGTTTTTTTCAGGATTAG
Core_08	ACTAGAAAAAGAATAATAATGG
Core_09	TTAGCGGGGTTTTCTGCAACTGT
Core_10	ATCAGGTCACGGTAATCCAACGC
Core_11	TGTAGCGTCAATCCAAATTCTTA
Core_12	TAACGACCGTGTGATAAGGGAAC
Core_13	CCTTTAATTGTATATTTTCCTGTG
Core_14	ATAGCAAACAGACACCCTGAGAGA
Core_15	GAACGTATGTAAATTTTTGATGCAAA
Core_16	GAGGCTAGGGCTTAATTTTTTATCGCC
Core_17	AACCGCTGCCAGCTTTTTTTTTTCCGGC
Core_18	AACTTGCCTTTAGCGTCTATTACGGAGGC
Core_19	AACAATTATCACCGTCAGACATTCGAAAA
Core_20	AGTATCGCCCACGCATAAGAAACCTGTAG
Core_21	ATAGAATAATAATTTTTTAAACAACGTAT
Core_22	GCAGCCTATTTCGGAACAAACAGTCCGGCGA
Core_23	TCAAGTTGGGAAAGTAATGCCCCCCTCCCTC
Core_24	TAAAAATTTAATGGTTTAATTTCAAAACTTT
Core_25	TATAGATGGCAATTTTTTTTCAATATAAACAGT
Core_26	TACCCCGCCAGCTTTTTTTGCAACAGGACCTGA
Core_27	CAGAGAGAATAACATAAAAGAGCAAGAAACAATG
Core_28	TCTAGACGGCCATTTTTTTCAAGCTTGCGTAAAG
Core_29	ATAGCTATCTTACCGAAGACGATTGGCCTTGATA
Core_30	CAAATAAATCCTCATTAGAACCCATGTACCGTAA
Core_31	GTAGATTTTTTTTTCAGTTTAAAATTTTTTTGAGTAA
Core_32	CGGGCTTTTTCTCTTCGTAACGCCTTTTTTTTTTTCC
Core_33	AAGGGTTTTTACATTCTAATACTTTTTTTTTGATTAG
Core_34	CACAATTTTTTTCCACAAAGCGGATTTTTTTATCAAA
Core_35	TCCAATCGCATGACCAACTTTGAAAGTTTTTTACAGAT
Core_36	CAAGAAATCGAGCCGGCTTGAGATGGTTTTTTTCAACTT
Core_37	CAACGCTGAAACGAATTAATTTTCCCTTAGAGGAATACCA

Core_38	CAGTATCCTTCTGGTCATAATCAAAATCTTTCGGTAATAA
Core_39	AGGCCGCTATAAAGCGTAAAACTAGCATCATACAAAGGCT
Core_40	AATATTATACATTTGGCCGGAAACGTCAGCCAGCAATTGC
Core_41	AGAGGGAATGCGCAATGGAAAGCGCAGCAGGGATGCAGGCG
Core_42	CCAGTTCAAAGCGCATGAGGAATTTGCCATCTTTTGCCGGA
Core_43	AAGCACTAAATAGAGTGAGGCCACCGATGCAACAAACGGAA
Core_44	CCAAAAAACCTTGGAAGCGCATTAGACAATTGAGGGTCAGT
Core_45	TTATCAAGAACCTAACGAAAGAGGCAAAAGCCTGCAGAAAA
Core_46	TTGCTGCTGAAATACGTAATGCTACCATTAGCAATGACGCT
Core_47	TGCCCGCTTTCTAGACTCCAACGTCAAGGCGCTACAGGAGT
Core_48	AATGCGCGAACTACTTTACAAACAATTCAATTTCAAAATTC
Core_49	AGAGTACCTTTCGAATGACCATAAATCTCTTTTCCCAGACG
Core_50	CAGACGCTCAAATTTAAGAAAAGTAAGACAGGAGTTGCTTT
Core_51	CAAGTTACAAACGGGGTTATATAACTAGTGTACAGTTACTT
Core_52	TCATTCAAACGAACTAAGCAATAAAGCCTTGATTCTCAGCTA
Core_53	TAGTTAGCATTACCACCTGTTTAGCTATCATCAATTTAAACA
Core_54	TCCGAAACCCGAGAAGTCCACTATTAAAGTAGCGGCATACAT
Core_55	ACCCTCACAATAATGTGCCACGCTGAGATATAATCCTAAACA
Core_56	GCCATATTAATTTGGACCTTCATCAAGAACTACCTTTTATCA
Core_57	GGAAACGGAGCCGCCAGAGCGGGCGGAACCCTAAAAGCCACC
Core_58	TGTCTGGGTTGTACGTCAGGACGTTGGGGAGAAACACCGACA
Core_59	CGCTAATTGAACAAGGAAACAGTACATAATACATTAATAGAT
Core_60	TTCCTCGGATTAAACTGAGAAGTGTTTTGCCAGCAAAACCGA
Core_61	ATGCAGAAAGTAATAGGCTTGCCCTGACAAGAAAAATAAAGC
Core_62	TAAATCGAAGTTTCTGTTTATCAACAATATAAAGTACCAGAA
Core_63	CCTTCACAAATGCTTCTACTAATAGTAGGTCAATAGCGCCCA
Core_64	CGAGTAGTATACCACAAAAACATTATGACAACTAATCCTGAA
Core_65	GCAAATGTAGCATTATTGAATCCCCCTCCGCCTGGGCCCTCA
Core_66	AAAGGTAACGCGCCATTCCATATAACAGTCAGAGCATCTACG
Core_67	TTAATAAGTGAATATCTGTCCAGACGACGACAATTAAGCTGC
Core_68	GAACCGCGATGATAGGGCGCTGGCAAGTGAACGTGAATCAAA
Core_69	GGAGGCCTTAGAATCACCAGAACCACCACAGAAGGGCAAATG
Core_70	GGCTTTTCACCCTCAATCCCTTACAGTCGGGAAACACCCTCA
Core_71	AGAATAGTCGGCAAAGAACCGCCACCCTTAAGCGTTCACGCT
Core_72	AAGGTGGATTTTCATTCATCGTAGGAATCGTAACGTGATTGC
Core_73	TTTATTTTGTGTTATCTAAAATGATAGCCCTAACGGAATAAG
Core_74	CGAAATCCGCAAAGATTAAGAATCGCGCAGAGGAAATTGTGT
Core_75	GCCCCAAGGAACGCCCTGATTATCAGATCTTCTGACACTAAA
Core_76	AGTTGCTTTCACCCAGCTACAATTTTTCAAAAATGAAAATAGC

Core_77	CCCCTTATTAGCGGTTTCACCAGTCACACCACTTGCACGACGA
Core_78	TCGTCATACAGCAAGGCCTGTAGCATTCCGCAAATCACATTTC
Core_79	CCAGTAGCACCATCAACGTAAAACAGAAATTTGCGGCCAGAAC
Core_80	TACCCAACCCTCAGGGGAGCCCCCGTTTTGAGCTTGACGTTTTG
Core_81	TTAGTAACATCGAGTGATAAGAGGTTTTTTTGCGGATGGACTCC
Core_82	ATATGGTGACACCAAACATCGCCATTTTTATACCGAACGTTTGA
Core_83	AGCCGGAGCCTGATCGAATTATTCATTTTATTACCTGAGTCGCC
Core_84	GTACTGGTACCGCCCTGTCGTGCCATTTTATTAATGAATCATTA
Core_85	CCTTTATTTATAATGCAATCAATAATCGGCAACAACCAATGTGT
Core_86	ACTGGCTCATTAAATTGAGTAATAAGAGAATAGATAAGAGTACGG
Core_87	ATTTAGTTTTTTGACCCATACAGTTTTTTGCAAAGAATTAGAGTAG
Core_88	TACTCTGAAACATGAAACTTGAGTGAAGGGAAGATTTTTTCGAAAG
Core_89	AGGTAAACAACATGTCCAATTCTGCGAACGCAAAATTAACGGAACAA
Core_90	TAATCATGATCTTCTGACTAACAACGCCAACATGTTTTTTAGGCAGA
Core_91	ACCGGGCCTCAGTTTTTTTATCGCACTCATGTGCTGCAAGACCCAAA
Core_92	CTCAATTTTTTTATGTTTTAAATATGCCCTGTAATACTTTGGGAGAAG
Core_93	CGTCGGGAAGCCCGAAGACAGTTTGCTTTCGAGGTGGCTCCAACCGCT
Core_94	CACGTTTTGTAAAACGAGGATCGCGGATAAGTGCCGAGAAGGACGGTG
Core_95	CAAGCGCCGACAATGACTGTCTTTCTTAATTGCTGAATCACGAACCAGA
Core_96	GAGCGAGGGCGAATTTTTCGTCTATCAACTACGTGGAGAAAGAACAGTG
Core_97	TATAGGAACAACTAAAGAGCGGAGCGCGCGGGGAGAGGAGTAATGAGTGA
Core_98	AGATTTGTCTGAGAGGTAATCTGTCTTTCCAGAGCCTATTTATTTGAAAA
Core_99	GAATTGAAACAACTTGAGGATTTAGAAGTTTTAATAGTCAGAGGCACAAT
Core_100	AAAACCACGGTACGCTAAAGCACCGAACAAAGTTACCCAGAGCACGTGCT
Core_101	AGTAAATCCAGTTTGACCACCAATTTACCGTTCCAGCAGAGCCTGGTGGT
Core_102	ACGAGGCAATAGATAAATCAATTTAACTGAACACCCATCAGAGTTGAAAG
Core_103	AAATCAGTAGCGACAGAACGTAGAAGCAGAAGATAAAAACACGTGGCACA
Core_104	ATCGGAAATTATTCATTGGGAAGGAGATGATGAAACAAACGGGAGAAACA
Core_105	ATATTCTACAAGAGATTTTTTATCGATGAATTGCCTGTTTTTTTTGTCTG
Core_106	GTTCAGACAACAGCATCTAAAGTTTTGTTTTTATTTTTGGGGCGAATTGC
Core_107	CATAGCGTAGTGAATTTTAACCTCCGGCGCTGGCTCCAGTTACAGACCTG
Core_108	CTCCTAGACTGTTTTTTTTCATCGGCATACCGGAACCAGAGTTTTCACCGG
Core_109	AAAAAGAATTTCTTAAACATTTTGATACCGAACGGGTATTTTTTTCAAGTA
Core_110	ATTAGACCAATGAAACCATTTTTAGCAGCAGGTGGCAACATTTTTAAAGAA
Core_111	GAAGGCTCTACAACCGGTCCACTTGCTGGTTTCCAATATTTTTTTTGCGGAA
Core_112	AGAGCCGCTTTTTCTCAGAACCGCCAAAGAACTGGCATGTTTTTTTTTAAGA
Core_113	GCTAACTCACGGCCAATGAGAATAGGAATAGGTGTATCACCTTTTTTCAGGAG
Core_114	ATAACGGATCAAAAGATAAATATTGGCGAAACAAAGTACAATTTTTGATTTGT
Core_115	CAGGTCAGCCCTTTATCAAACCTTCTCAATCTCATAACTTTTTTTTTCGTTTAC

Core_116	GAGCTGTTAAAATTCGAGAACGCGATGTGAATTACCTTATTTTTTTTTTTAAGA
Core_117	AATGGGTCGAATTCTACAGAGGGCTCAGTACCAGCCCGGGTACCGAGCATAGGT
Core_118	CGAGTATATGATAAAAGACAGCCGGTTTATCAGCCAAATCACCATCAAACAACC
Core_119	GTTTAGTAATAAGTTTTAATTTTTTTGGGGTCAGTGCGTATTATTTTGAGACTC
Core_120	CCAATAGAAGCCAGCGCTACAGTTGGCGCGTGCAAAAGTTTTTTTTGTTTTGCC
Core_121	CAATAATAAACAGGCTTCTGTATTAATCGTCAATGCAGTTTTTTTTACATAACG
Core_122	ATCATCACGAGGCGCAGACTTTTTTTGTCAATCATAAATAAGGTTTTTAAGAAT
Core_123	GAGGGTAGTTTTTTCAACGGCGTAATCTTATGGTCGGCGGATTTTTTTTGACCGT
Core_124	AAATCATAGAATCCCCCAATCCAAATAAAACGAGCTGACAAGAACCGGACAGTTG
Core_125	TAAACGGGTTTTTTTAAAATGGATTATTTTTACATTCAAACTTTTTTTATCGGCC
Core_126	CATTCAACTGCTATTATTTTTTGTTTAACGATCCTGATACCCAAATTTTTCAACG
Core_127	CAATAGATTTGAATTACCTTTATTAGATCTTTAGGAGCACTGGAAGGTAATTGAG
Core_128	TCCTTTAACAAGCAAGCCGTCGTCTTTACCAGTGAGACGGGAAACGAGAGCTGAA
Core_129	ACGCAAATTACCAGCGCCAATTTTTTTGACAAAAGGGCCCGACTTTTTTTTGGGA
Core_130	TAGAGCCTCAACAGAGATAACCCACAAGGGGAGAAATATGTGAGTGAATGGAATT
Core_131	GAGTGTTTGTTTGAACCACCCTCATTTTTCTCTGACACCCGCCGCGCTTGGTAAT
Core_132	AGGCACCATTTTTTACCTAAACCATATTTCAAAATCAGAAGGTTTTTTAGCGGAA
Core_133	CTCCATGACCAGGCGCATAGTTAGGTTCTGAGAAGAGTCAAATAGCTTATAAACA
Core_134	TAGACAGACGTATACGCCGCCAGCATTGCAGATAGTCACCTTGCTGAACACGATA
Core_135	ACTAAAGATTTTTTCTTTTTCCATTCGTTCCATTCTAACCGTTTTTTTGCATCTG
Core_136	CCGCACTATGAATTTTCTGTTTTTTTTTGGGATTTTGCTCACGTTTTTCTCCAAA
Core_137	GGCATTTAATAATATCCCATCCTTTTTTTACGAGCATGTACCGATTTTTGGTCGCT
Core_138	CAAAATTTTTTATTACATTTAACGACAACTTTTTTTTAAATCCTTTGCCCGAACAT
Core_139	TCAACAGTTGCAGGTATTTTAAATTTTTTGCAATGTAAAAATTTTTTTGAACCCT
Core_140	TGGGCTTTTTTAGGGTGGTTTTAAAAATCATTTTTTTTACCCTGACTATTATCGGT
Core_141	GGTCATTTTTTTTAACACCGCCGTAAAAGATTTTTGTCCATCACGCAAATTACAGA
Core_142	ACACTCACGGAATCAAGATTGTATTTTTTAAGCAAATAAATCATTTTTTTATTTTT
Core_143	AAACACTCTTTGACCCCCATTTTTTATACCAAGCACCTGATTGTTTTTTTTTTGGAT
Core_144	CTCAAGTCGAGAGGGTTGATTTTAGTATAGCCCGAAGCCTGCAGTTTTTTTGTCGAC
Core_145	CACCCTCATTTTTTGCAGCGTTCAACCTTGTTCTAGAACATTAATTTTTTATGTGAG
Core_146	TTCGTCACCAGTACAAATATCCGGTATTCTAAGATTGTTTTAGCGAACCTCAAGATT
Core_147	ATCATATGTTTTTTCGTTATAATATGTACTTCCCGGTCGCGTCTTTTTTTGGCCTTCC
Core_148	GACAATATTAACCACCAAATACATACATAAACCGTAAAACGCTCATTTTTTTTGGAAA
Core_149	CCGGAAGCAACTTAGAGCCTTATCATTCCAAGATAGTTAAGGGTGATTTTTTTGAAAGG
Core_150	TAACATTGAAGCCTTAAATCCCGACTTGCGGGCCGAAAATCAAGTGAGCG
Core_151	GTTGAATATTCAACATCCAATAAATATTAGATAGATATACCGAAAATCAAGTGAGCG
Core_152	AAAATCCGTATGTTTAGACTGGATAGCGTGCCCCAAGCAAGCCCGAAAATCAAGTGAGCG
Core_153	CATTATTTTTTTTTGTAGAAAGATTCATTATTCATATCTTACCCGAAAATCAAGTGAGCG
Core_154	GACGAGCGAAAAATAGCGAGAGGCTTTTACTATGGGTTGAGGCCGAAAATCAAGTGAGCG

Core_155	TGGCAAAGTCATAGTAAGAGCAACACTAAATATCTTTAAGCCCCGAAAATCAAGTGAGCG

Table S2. Extended staples that serve as a docking site for sc-TRAIL.

Ext_Staple_DNA_01	CAGTCACGAGGGCGATTTAGGAGTGCGCAAAGAGTTTAGC
Ext_Staple_DNA_02	TAACCAATAAAACAGGATAATTGTGCGCAAAGAGTTTAGC
Ext_Staple_DNA_03	TAATAACATGACCAGTCATAGCGTGCGCAAAGAGTTTAGC
Ext_Staple_DNA_04	AAGATTAAGTTGTTATAAGGAGGTGCGCAAAGAGTTTAGC
Ext_Staple_DNA_05	CATTATCATTAAAGAAAAATCAGTGCGCAAAGAGTTTAGC
Ext_Staple_DNA_06	CCTGGGGTGCCTCAGAAGCACAACGTGCGCAAAGAGTTTAGC
Ext_Staple_DNA_07	AAGCGTAAGAATCGTTGTAGCGGCCGTGCGCAAAGAGTTTAGC
Ext_Staple_DNA_08	ACCTTTTACATCGTTATTAATGTTTGTGCGCAAAGAGTTTAGC
Ext_Staple_DNA_09	AGGTAAACGAGCTTCAAAGACGCAAGGACCTGGTGCGCAAAGAGTTTAGC
Ext_Staple_DNA_10	ACGTGGCAACCATCGCGATTAAGTTGGGCTATGTGCGCAAAGAGTTTAGC
Ext_Staple_DNA_11	CCCGTATCTATTATGCCAGCTGGCGAAAGGGGGTGCGCAAAGAGTTTAGC
Ext_Staple_DNA_12	CCGGAAGACTTCTTTTTTTATCGCGTTTTAATTGATTGTGCGCAAAGAGTTTAGC
Ext_Staple_DNA_13	CCAGTTTTGCGATTAATGCCGGAGAGGGTATAGAGTTAAGTGCGCAAAGAGTTTAGC
Ext_Staple_DNA_14	TGAAAAGATTCTCCGTGGGAACAAACATAGCTGCGGAACGTGCGCAAAGAGTTTAGC
Ext_Staple_DNA_15	TGGGAACGGGTGTAGATGGGCGCATCGAGGCTGCTTGAGGGTGCGCAAAGAGTTTAGC
Ext_Staple_DNA_16	AACCAGGTGAGGGGCTGAGTAGAAGAACTGGCAGATTCCATGTGCGCAAAGAGTTTAGC
Ext_Staple_DNA_17	CAATCGTGTAATATAACAAAGAAACCACTATTTGCCTACGAGTGCGCAAAGAGTTTAGC
Ext_Staple_DNA_18	AAGGGTTTCATATTCATCAAAAATAATTTGATAATTTTAGTGTGCGCAAAGAGTTTAGC
Ext_Staple_DNA_19	CAAAACCGATTGAGGGAAAAGGTGGTCAGATGAATATACAGTGTGCGCAAAGAGTTTAGC
Ext_Staple_DNA_20	TTGCTTTCAACAGTTTCGAATTGCCGAGCCGGAAGCATAAAGGTGCGCAAAGAGTTTAGC
Ext_Staple_DNA_21	GGTCAGTATGTTAGCAAATCAAGTAGAGATAGAACCCTTCTGGTGCGCAAAGAGTTTAGC
Ext_Staple_DNA_22	CAGCTATTTTTGAGAGATCGCTTTCATCCTGATAAGGATCGTGTGCGCAAAGAGTTTAGC
Ext_Staple_DNA_23	TTCAAATAAGACAACATTAAATTTTTGTTATTGTGCGCAAAGAGTTTAGC
Ext_Staple_DNA_24	CGAACTATTTTAGTTGAAATACATTGTAAACGTTAATATTGTGCGCAAAGAGTTTAGC
Anti_Ext_Staple	/5AmMC6/GTGCTAAACTCTTTGCGCAC

Table S3. Unextended staples that replace the extended staples. *Combination of different extended/unextended staples allows docking of different amounts of sc-TRAIL or with different spacing on the DNA origami nanostructure.*

Unext_Staple_01	CAGTCACGAGGGCGATTTAGGA
Unext_Staple_02	TAACCAATAAAACAGGATAATT
Unext_Staple_03	TAATAACATGACCAGTCATAGC
Unext_Staple_04	AAGATTAAGTTGTTATAAGGAG
Unext_Staple_05	CATTATCATTAAAGAAAAATCA

Unext_Staple_06	CCTGGGGTGCCTCAGAAGCACAAC
Unext_Staple_07	AAGCGTAAGAATCGTTGTAGCGGCC
Unext_Staple_08	ACCTTTTACATCGTTATTAATGTTT
Unext_Staple_09	AGGTAAACGAGCTTCAAAGACGCAAGGACCTG
Unext_Staple_10	ACGTGGCAACCATCGCGATTAAGTTGGGCTAT
Unext_Staple_11	CCCGTATCTATTATGCCAGCTGGCGAAAGGGGC
Unext_Staple_12	CCGGAAGACTTCTTTTTTTATCGCGTTTTAATTGATT
Unext_Staple_13	CCAGTTTTGCGATTAATGCCGGAGAGGGTATAGAGTTAA
Unext_Staple_14	TGAAAAGATTCTCCGTGGGAACAAACATAGCTGCGGAAC
Unext_Staple_15	TGGGAACGGGTGTAGATGGGCGCATCGAGGCTGCTTGAGG
Unext_Staple_16	AACCAGGTGAGGGGCTGAGTAGAAGAACTGGCAGATTCCAT
Unext_Staple_17	CAATCGTGTAATATAACAAAGAAACCACTATTTGCCTACGA
Unext_Staple_18	AAGGGTTTCATATTCATCAAAAATAATTTGATAATTTTAGT
Unext_Staple _19	CAAAACCGATTGAGGGAAAAGGTGGTCAGATGAATATACAGT
Unext_Staple _20	TTGCTTTCAACAGTTTCGAATTGCCGAGCCGGAAGCATAAAG
Unext_Staple _21	GGTCAGTATGTTAGCAAATCAAGTAGAGATAGAACCCTTCTG
Unext_Staple_22	CAGCTATTTTTGAGAGATCGCTTTCATCCTGATAAGGATCGT
Unext_Staple_23	TTCAAATAAGACAACATTAAATTTTTGTTATT
Unext_Staple_24	CGAACTATTTTAGTTGAAATACATTGTAAACGTTAATATT

Table S4. Combinations of extended/unextended staples used for different DNA origami nanostructures with different valencies and spacing.

Construct	# Extended staples	# Unextended staples
Ori-0	None	All 24
Ori-1	22	1-2-3-4-5-6-7-8-9-10-11-12-13-14-15-16-17-18-19-20-21-23-24
Ori-2	18-22	1-2-3-4-5-6-7-8-9-10-11-12-13-14-15-16-17-19-20-21-23-24
Ori-3	14-18-22	1-2-3-4-5-6-7-8-9-10-11-12-13-15-16-17-19-20-21-23-24
Ori-4	14-15-18-22	1-2-3-4-5-6-7-8-9-10-11-12-13-16-17-19-20-21-23-24
Ori-5	14-15-16-18-22	1-2-3-4-5-6-7-8-9-10-11-12-13-17-19-20-21-23-24
Ori-6S	14-15-16-17-18-22	1-2-3-4-5-6-7-8-9-10-11-12-13-19-20-21-23-24
Ori-6M	1-2-3-4-5-13	6-7-8-9-10-11-12-14-15-16-17-18-19-20-21-22-23-24
Ori-6L	6-7-8-9-10-23	1-2-3-4-5-11-12-13-14-15-16-17-18-19-20-21-22-24
Ori-12	1-2-3-4-5-13-14-15-16-17-18-22	6-7-8-9-10-11-12-19-20-21-23-24
Ori-24	All 24	None

Table S5. Sc-TRAIL amino acid sequence

MGPQRVAAHITGTRGRSNTLSSPNSKNEKALGRKINSWESSRSGHSFLSNLHLRNG
ELVIHEKGFYYIYSQTYFRFQEEIKENTKNDKQMVQYIYKYTSYPDPILLMKSARN
SCWSKDAEYGLYSIYQGGIFELKENDRIFVSVTNEHLIDMDHEASFFGAFLVGGGS
GGSGGSPQRVAAHITGTRGRSNTLSSPNSKNEKALGRKINSWESSRSGHSFLSNLH

```
LRNGELVIHEKGFYYIYSQTYFRFQEEIKENTKNDKQMVQYIYKYTSYPDPILLMK
SARNSCWSKDAEYGLYSIYQGGIFELKENDRIFVSVTNEHLIDMDHEASFFGAFLV
GGGSGGSGGSPQRVAAHITGTRGRSNTLSSPNSKNEKALGRKINSWESSRSGHSFL
SNLHLRNGELVIHEKGFYYIYSQTYFRFQEEIKENTKNDKQMVQYIYKYTSYPDPI
LLMKSARNSCWSKDAEYGLYSIYQGGIFELKENDRIFVSVTNEHLIDMDHEASFFG
AFLVGGGSGGSGGSLPETGGHHHHHH
```

Methods

Design, folding and purification of DNA origami nanostructures

The DNA origami nanostructures were designed using Athena,[2] using ssDNA m13mp18 viral genome as the scaffold.M13mp18 ssDNA viral genome was ordered from Tebubio. Molecular graphics and analyses were performed with UCSF Chimera/ChimeraX, developed by the Resource for Biocomputing, Visualization and Informatics at the University of California, San Francisco.[3] The DNA origami nanostructures and the staples were modified using cadnano2.[4] All used strands were ordered from Integrated DNA Technologies (IDT), Leuven, Belgium. After identification of conjugation sites on the nanostructure, each staple at that location was extended with the sequence GTGCGCAAAGAGTTTAGC. These extended staples could then be exchanged with unmodified staples to select specific sites for conjugation.

In the folding reaction mixture, the final concentration of the scaffold DNA and the oligonucleotide (IDT) strands were 20 nM and 200 nM, respectively with a final volume of 50 µL. As determined empirically (**Figs. S1A, B**) the folding reaction buffer contained 20 mM Tris, 20 mM NaCl and 14 mM $MgCl_2$. The folding reactions were thermally annealed using C1000 Touch Thermal Cycler (Biorad). The reactions were left at 80°C for 1 minute, followed by a thermal annealing ramp from 80°C to 75°C (0.2°C/min), then subsequently from 75°C to 30°C (0.1°C/min) and finally from 30°C to 20°C (0.1°C/min). The folded structures were purified from excess oligonucleotides using the Amicon Ultra 0.5 – 100 kDa filters and the staples were diluted around $10^{\wedge 6}$ times.

Transmission electron microscopy sample preparation and image acquisition

10 µL of DNA nanostructureswere applied at 2 nM to glow-discharged C-flat carbon film 200 mesh grids (Aurion, Netherlands) and incubated for 1 minute. After removal of the excess solution, the staining was performed with 10 µL of 2% uranium formaldehyde (SPI-Chem, 02545-AA) and incubated for 10 seconds. After removal of the solution the grid was air dried for 5-15 minutes. Images were obtained using a Tecnai T12 Biotwin transmission electron microscope operated at a voltage of 120 kV.

CryoEM sample preparation, image acquisition and single-particle analysis

T-hex origami sample was used at a concentration between 1-2 µM in 20 mM Tris, 20 mM NaCl and 14 mM $MgCl_2$ buffer. For cryo-EM grid preparation multiple conditions were tested, with an optimal particle distribution obtained using 3.5 µl of T-hex origami sample added to glow discharged Quantifoil grids with a 300 mesh copper support and holes sizes of 2/1 µm. Grids were blotted for 1 seconds with Whatman No. 1 filter paper using a Leica EMGP with a chamber environment at 10°C and 85% humidity, before plunge freezing in liquid ethane.

Cryo-EM movies were collected on a Talos Arctica microscope (ThermoFisher) at the Electron Microscopy Facility of Leiden University Medical Center operated at 200 keV and equipped with a Gatan K3 detector and Gatan BioQuantum K3 energy filter with a slit width of 20 eV. Movies were acquired using a total dose of 60 e/Å2 with 50 frames, at 79000× magnification with a calibrated pixel size of 1.08 Å. Motion correction and CTF estimation of the micrographs were performed using Relion 3.1.[5] Initially, particles were manually picked to generate 2D templates for subsequent template-based template/matching autopicking resulting in 21000 particles. Particles were binned by 4× and subjected to 2D classification and only the 2D classes representing the structure of interest were selected for further steps. An initial model was generated with C6 symmetry applied. 3D classification was performed to remove badly aligned particles. Particles were polished and final refinement and postprocessing were performed, resulting in a final map at 45 Å resolution (**Fig. S2**).

Production and purification of sc-TRAIL in BL21 Rosetta2

E. coli Rosetta2 (DE3) (Novagen) was transformed with pCPF3.05 encoding for sc-TRAIL. Sequence of sc-TRAIL is given **Table S5**. Protein production was initiated with 1 mM isopropyl β-D-1-thiogalactopyranoside (IPTG) (VWR, 437145X) and the culture was supplemented with 100 µM $ZnCl_2$ (MERCK, 96468) or $ZnSO_4$ (MERCK, 221376). Incubation took place at 18°C 200 rpm for about 24 hours, after which the protein was purified with 500 µL of HisPur™ Ni-NTA Resin from Thermo Fisher Scientific. The proteins were first washed 3 times with 10 mL washing buffer containing 100 mM Tris pH 7.5, 150

mM NaCl, 100 µM $ZnCl_2$, 10% glycerol and 50 mM imidazole. Afterwards, the proteins were eluted with 4 mL elution buffer containing 100 mM Tris pH 7.5, 150 mM NaCl, 100 µM $ZnCl_2$, 10% glycerol and 250 mM imidazole. The eluted proteins at 250 mM imidazole were used for the performing the transpeptidation reaction with Sortase 5M.

Transpeptidation reaction of sc-TRAIL to functional peptides using Sortase 5M

Conjugation was performed by incubating 50 µM sc-TRAIL, 1 mM functional peptides containing FITC or azide, 25 µM sortase 5M, in a buffer containing 10 mM $CaCl_2$, 50 mM TRIS, 150 mM NaCl and 10% glycerol, pH 7.5 with a final volume between 0.5-1 mL. The reaction mixture was incubated at 4°C, mixing for total of 2 hours. Followed by further purification using ÄKTA™ pure micro with HiLoad® 16/600 Superdex® 75 pg with 1 ml/min. Elutions containing the sc-TRAIL were then concentrated using 15 mL Amicon Ultra-15 50 kDa (30 minutes, 3220 rcf, 4°C) if necessary and the click reaction was performed. The sequence of the functional peptides are as follows: NH_2-GGG-K(FITC)-G-H and NH_2-GGG-K(N_3). Martijn Verdoes (Radboud University) kindly gifted the plasmid encoding for Sortase 5M and the azide peptides, FITC containing peptides were acquired from Peptide Facility, CCB, LUMC, Netherlands.

Production and purification of Sortase 5M

E. coli (DE3) (Novagen) was transformed with pET30b encoding for Sortase 5M.[6] This plasmid can be found on Addgene with the plasmid number #51140. Protein production was initiated with 1 mM isopropyl β-D-1-thiogalactopyranoside (IPTG) (VWR, 437145X). Incubation took place at 18°C 200 rpm for about 24 hours, after which the protein was purified with 500 µL of HisPur™ Ni-NTA Resin from Thermo Fisher Scientific. The proteins were first washed 3 times with 10 mL washing buffer containing 100 mM Tris pH 7.5, 150 mM NaCl, 10% glycerol and 50 mM imidazole. Afterwards, the proteins were eluted with 4 mL elution buffer containing 100 mM Tris pH 7.5, 150 mM NaCl, 10% glycerol and 250 mM imidazole. The eluted proteins at 250 mM imidazole were further purified using ÄKTA™ pure micro with HiLoad® 16/600 Superdex® 75 pg with 1 ml/min. Elutions containing the Sortase 5M were then concentrated using 15 mL Amicon Ultra-15 10 kDa (30 minutes, 3220 rcf, 4°C) if necessary.

Click reaction

Dibenzocyclooctyne-N-hydroxysuccinimidyl (DBCO) ester (Sigma Aldrich, 761524) was used to DBCO functionalize the DNA handles containing an amine group. 1.5 mg of DBCO ester was dissolved in 300 µL DMSO. DNA handles were DBCO functionalized by mixing 20 µL DBCO ester, 20 µL DMSO, 20 µL PBS and 20 µL amino modified DNA handles and incubating at 37°C, 1100 rpm overnight. DBCO-modified DNA

handles were purified using Amicon Ultra-0.5 10 kDa filters (10 minutes, 13000 RCF, RT) and the concentration was determined with the absorbance at 260 nm.

sc-TRAIL-N_3 was incubated with DBCO containing DNA handles at 4°C overnight with 3 µM ligand and 30 µM oligo concentrations. sc-TRAIL-DNA was then purified from the excess DNA-DBCO using Amicon Ultra-0.5 50 kDa (10 minutes, 3000 rcf, RT). The reaction was monitored with SDS-PAGE Bolt™ 4 to 12%, Bis-Tris, 1.0 mm from Thermo Fisher Scientific with a running scheme of 200 V for 35 minutes. Proteins were stained with SimplyBlue™ SafeStain from Thermo Fisher Scientific.

Decoration of DNA origami nanostructures with sc-TRAIL-DNA

Equimolar concentrations of sc-TRAIL-DNA was incubated with 35 nM DNA origami nanostructures at RT overnight followed by purification with Amicon Ultra-0.5 100 kDa filters (4 minutes, 3000 rcf, RT). The concentration was determined by absorbance at 260 nm.

Cell culture and cell viability assay

Jurkat (Mirjam Heemskerk, LUMC, the Netherlands) and JeKo-1 cells (Eric Eldering, AMC, the Netherlands) were cultured in IMDM medium (Thermo Fisher, 12440053) and RPMI-1640 (Thermo Fisher, 11875093), respectively. Both media were supplemented with 10% FBS (ThermoFisher, A5209401), 1x Glutamax (Invitrogen), 100 IU/mL penicillin and 100 µg/mL streptomycin. Both cell lines were seeded at 6.67×10^5 cells/mL in a 96-well plate 5 overnight at 37°C, 5% CO_2 at a final volume of 45 µL. Cells were treated with different concentrations of 5 µL sc-TRAIL containing DNA origami nanostructures with a final culture volume of 50 µL and incubated at 37°C, 5% CO_2 overnight. Cell viability was determined by adding 7.5 µL dye from CellTiter 96® Non-Radioactive Cell Proliferation Assay (MTT) from Promega and was incubated for 3 hours at 37°C, 5% CO_2. The reaction was stopped by adding 50 µL of stop solution. The plate was incubated overnight at 37°C, 5% CO_2 to help dissolve the crystals.

Patient-derived organoid cultures

The study was approved by the medical ethics Committee of Leiden University Medical Center (reference numbers RP24.004 and B22.036). Primary organoid cultures were established from human pancreatic cancer or colorectal cancer tissue obtained during surgical resections in accordance with the Code of Conduct for Responsible Use of Human Tissues, after written informed consent was obtained. Organoids were cultured in growth factor–reduced, phenol red–free Matrigel (Corning, NY) domes overlaid with organoid growth medium (below) and passaged by mechanical dissociation using a 1 mL syringe.

The medium for PDAC organoids consisted of advanced DMEM/F12, 1x Glutamax (Invitrogen), 100 µg/mL Primocin (Bio-Connect), HEPES (Life Technologies), 1x B27 supplement (Invitrogen), 5 mM Nicotinamide (Sigma), 10 µM ROCK inhibitor Y-27632 (Bio-Techne, Minneapolis, MN), 10 ng/ml Wnt3a (Biolegend), 100 ng/mL recombinant Noggin (Peprotech), 1 µg/ml recombinant R-Spondin 1 (Peprotech), 1 mM N-Acetyl Cysteine (Sigma), 500 nM A83-01 (Selleckhem), 10 nM Gastrin (Sigma), 20 ng/mL recombinant fibroblast growth factor-10A (Peprotech), 50 ng/mL recombinant epidermal growth factor (Peprotech).

The medium for CRC organoids was advanced DMEM/F12, supplemented with 1x Glutamax (Invitrogen), 100 IU/mL penicillin and 100 µg/mL streptomycin, 100 ng/mL recombinant R-spondin 1 (Peprotech), 100 ng/mL recombinant Noggin (Peprotech), 10 nM Gastrin (Sigma), 100 ng/mL recombinant epidermal growth factor (Peprotech), 0.5 nM A83-01 (Selleckhem), 100 µg/mL Primocin (Bio-Connect), 1.25 mM N-Acetyl Cysteine (Sigma), 10 mM Nicotinamide (Sigma), and 1x B27 supplement (Invitrogen). Media were changed every 2–3 days.

3D Cell viability assay

To examine the viability of organoids upon different treatments, CellTiter-Glo® 3D Cell Viability Assay (Promega, The Netherlands) was performed according to the manufacturer's protocol. Briefly, PDAC or CRC organoids were seeded at 3000 cells in a 20 µl Matrigel dome per well (3 wells per condition) in 48-well plates and overlaid with their respective culture media. After 3 days, once the organoids had formed, the growth media were removed and replaced with medium containing varying concentrations of scTRAIL or Ori-6S for 4 days. Growth medium only, origami only, and unconjugated TRAIL were used as controls. Organoids were imaged daily for 4 days using the Cytation Microplate Reader (Biotek, USA) and cell viability was assessed on day 4. In short, 50 µl of reagent per well was mixed with 100 µl of fresh organoid medium and added to each well. After incubation for 30 min, luminescence was measured using a SpectraMax® iD3 microplate reader (Molecular Devices LLC, USA). The percentage of cell viability was calculated by dividing the luminescence values of the treated wells by those of the medium control.

3D Apoptosis assay

In 48-well plates, organoids were seeded at 3,000 cells in a 20 ul dome per well and cultured for 3 days. Next, the organoids were treated with either growth medium only, origami only, unconjugated TRAIL, 30 nM scTRAIL or 30nM Ori-6S. To measure caspase 3/7 activity, the CellEvent Caspase 3/7 Green Detection assay (Invitrogen, The Netherlands) was performed following the manufacturer's protocol. Caspase 3/7 activity was monitored using a Leica SP8 white light laser confocal microscope (Leica Microsystems,

Germany) at 500 nm excitation and 530 nm emission, with Z-stack images taken at 1h intervals throughout the experiment.

References

(1) Aba, G.; Scheeren, F. A.; Sharp, T. H. Design and Synthesis of DNA Origami Nanostructures to Control TNF Receptor Activation. *Methods Mol Biol* **2024**, *2800*, 35-53. DOI: 10.1007/978-1-0716-3834-7_4 From NLM.

(2) Jun, H.; Wang, X.; Parsons, Molly F.; Bricker, William P.; John, T.; Li, S.; Jackson, S.; Chiu, W.; Bathe, M. Rapid prototyping of arbitrary 2D and 3D wireframe DNA origami. *Nucleic Acids Research* **2021**, *49* (18), 10265-10274. DOI: 10.1093/nar/gkab762 (acccessed 1/10/2025).

(3) Meng, E. C.; Goddard, T. D.; Pettersen, E. F.; Couch, G. S.; Pearson, Z. J.; Morris, J. H.; Ferrin, T. E. UCSF ChimeraX: Tools for structure building and analysis. *Protein Sci* **2023**, *32* (11), e4792. DOI: 10.1002/pro.4792 From NLM.

(4) Douglas, S. M.; Marblestone, A. H.; Teerapittayanon, S.; Vazquez, A.; Church, G. M.; Shih, W. M. Rapid prototyping of 3D DNA-origami shapes with caDNAno. *Nucleic Acids Res* **2009**, *37* (15), 5001-5006. DOI: 10.1093/nar/gkp436 From NLM.

(5) Scheres, S. H. W. RELION: Implementation of a Bayesian approach to cryo-EM structure determination. *Journal of Structural Biology* **2012**, *180* (3), 519-530. DOI: https://doi.org/10.1016/j.jsb.2012.09.006.

(6) Shi, J.; Kundrat, L.; Pishesha, N.; Bilate, A.; Theile, C.; Maruyama, T.; Dougan, S. K.; Ploegh, H. L.; Lodish, H. F. Engineered red blood cells as carriers for systemic delivery of a wide array of functional probes. *Proceedings of the National Academy of Sciences* **2014**, *111* (28), 10131-10136. DOI: 10.1073/pnas.1409861111 (acccessed 2024/10/23).
